# Intersectoral Collaboration Among Community Health and Social Workers in Disability-Related Organizations in South Korea: Awareness, Perceived Importance, Frequency, and Satisfaction

**DOI:** 10.5334/ijic.8583

**Published:** 2025-07-07

**Authors:** Hye-Jin Kim, Jae-Young Lim, Soong-Nang Jang

**Affiliations:** 1 Chung-Ang University, Seoul, Republic of Korea; 2 Department of Rehabilitation Medicine, Seoul National University College of Medicine, Seoul National University Bundang Hospital, Gyeonggi-do, Korea

**Keywords:** Intersectoral collaboration, Disabilities, Importance-Performance analysis, Care coordination, Integrated care

## Abstract

**Introduction:**

Disability research and policy emphasize cross-sector collaboration due to the complexity of disability. This study aimed to assess service providers’ awareness of disability-related community resources and evaluate their perceptions of the importance, frequency, and satisfaction with intersectoral collaboration.

**Methods:**

A cross-sectional study was conducted in Gyeonggi Province, Korea, using a mixed-methods design. The quantitative component examined community health and social workers’ awareness, perceived importance, frequency, and satisfaction with intersectoral collaboration. Qualitative data were collected through an open-ended survey question and analyzed using content analysis.

**Results:**

Findings revealed low awareness of medical and public health agencies, particularly newly designated disability-focused organizations in Korea, and infrequent collaboration despite its recognized importance. Differences in perceived importance and collaboration frequency across sectors were visualized using the IPA grid. Six key themes emerged from the qualitative analysis: ‘Information,’ ‘Governance,’ ‘Administrative processes,’ ‘Workforce,’ ‘Resources,’ and ‘Clients.’

**Discussion:**

The differences and commonalities in service providers’ perceptions, depending on their sector affiliation, highlight opportunities for policymakers to implement strategies that foster cross-sector partnerships.

**Conclusions:**

This study underscores the role of awareness and perceived importance in fostering cross-sector collaboration, offering insights for future policies and initiatives aimed at improving collaboration in disability care.

## Introduction

Integrated care has been a central focus in health and social care policies for decades, aiming to address complex care needs with a theoretical foundation rooted in institutional, stakeholder, or resource dependence perspectives [1]. The integrated approach seeks to mitigate challenges arising from a fragmented care system, particularly in the context of an aging society with increasing multimorbidity—issues that individual health and social care providers cannot address in isolation [1,2,3]. The concept of integrated care is broadly defined, encompassing attributes like person-centered care, transitional care, coordination of care services, continuity of care, case management, and interdisciplinary or inter-organizational cooperation [2,4]. Disability research and policy, in particular, emphasize intersectoral collaboration, wherein multiple organizations play distinct roles and coordinate their efforts toward a common goal [5,6,7]. This approach is especially important for people with disabilities (PWDs), who are highly diverse, spanning all ages and types of functional limitations, and are disproportionately affected by social determinants of health [8,9]. PWDs often experience multimorbidity, including secondary conditions arising from primary impairments, functional limitations, or additional disabilities. As a result, they are particularly vulnerable to health and healthcare-related social determinants—such as limited access to healthcare, employment, education, and public transportation—requiring integrated approaches across multiple service sectors, including health, housing, social, and education systems, to effectively address their complex needs and improve overall well-being [8,10,11,12].

Despite the widespread consensus on the necessity of intersectoral collaboration, empirical evidence highlights significant gaps in its implementation. For example, an evaluation of the Korean Healthy Cities network revealed rare collaboration with other departments within municipalities, with activities mostly limited to supportive roles, such as providing venues and publicity [13]. Previous studies have identified barriers to effective collaboration, including differences in agendas and priorities, lack of mutual understanding, organizational recognition, cultural distance, unshared values between sectors, and issues related to the structure, constitution, and processes of partnerships [4,6,13,14,15,16,17]. From the perspective of service providers, difficulties in sharing confidential information, a lack of understanding of health issues [18], and the perceived importance of collaboration are also significant challenges [16].

Frontline service providers, especially community health and social workers for PWDs, often face complex health and social problems that cannot be addressed single-handedly. These workers are recommended to work across traditional service boundaries, establishing cross-sectoral relationships to meet care needs [1,19]. However, these workers frequently encounter difficulties in building partnerships with non-disability-inclusive communities, compounded by a fragmented health and social care system. The lack of cross-sector collaboration contributes to fragmented service provision, acting as a barrier to service access [20]. Global policies, including the community-based rehabilitation model, emphasize disability-inclusive strategies and intersectoral collaboration to address health inequities [19,21,22]. Some disability studies have explored intersectoral collaboration, such as the collaborative practices for Aboriginal and Torres Strait Islander childhood disability, which were influenced by factors like shared goals, vision, and coordinated governance [20]. Similarly, studies on collaboration between disability and palliative care services for people with intellectual disabilities highlighted the lack of mutual understanding as a barrier [23]. Across various sectors, many studies emphasize poor organizational recognition and mutual understanding, including the perceived importance of cooperation, as major obstacles to intersectoral collaboration.

In response to international policy recommendations, the Korean government has made significant strides in establishing disability-inclusive initiatives since ratifying the Convention on the Rights of Persons with Disabilities in 2008. A key effort was the enactment of the Act on Guarantee of Right to Health and Access to Medical Services for Persons with Disabilities (hereafter referred to as the Act on Right to Health for PWDs) in 2017, which led to various public health initiatives aimed at improving healthcare access and reducing health disparities among PWDs [24]. These initiatives include the primary healthcare pilot program, the designation of disability-friendly medical checkup facilities, and the establishment of Regional Health and Medical Centers for PWDs (RHMCs). The primary healthcare pilot program incorporates team-based healthcare and home-based primary care to enhance healthcare accessibility, continuity, and sustainability for individuals with disabilities [25]. Notably, nationwide, RHMCs serve as hubs within a fragmented healthcare system by integrating healthcare services and bridging gaps between community healthcare and social care systems. They play a crucial role in identifying community resources, fostering partnerships, and connecting PWDs to appropriate services to address their comprehensive needs [26].

Given Korea’s significant efforts to establish disability-health partnerships and enhance intersectoral collaboration, this study aims to examine community health and social care workers’ awareness of disability-related community resources, assess the perceived importance, frequency, and satisfaction with intersectoral collaboration, and explore their perceptions of collaboration. Although a few prior studies defined a “community health worker” as a frontline public health worker, not a licensed professional [27], in this study, the term “community health workers and social workers” included all licensed professionals, such as physicians, nurses, physiotherapists, occupational therapists, social workers, and health administrators in clinical or public health or social welfare settings. In the context of intersectoral collaboration within a local community, healthcare providers should collaborate as community members, even in clinical settings, regardless of whether they serve outpatients or inpatients.

## Methods

### Study design and sample

A mixed-methods design was employed, integrating both quantitative and qualitative approaches. The quantitative component examined community health and social workers’ awareness of community organizations, as well as their perceived importance, frequency, and satisfaction with intersectoral collaboration. To gain deeper insights into their perceptions of intersectoral collaboration, qualitative data were collected through an open-ended survey question and analyzed using content analysis.

This study was conducted at the Gyeonggi RHMC for PWDs which was funded and designated by the Korean government. Gyeonggi Province has the largest registered disability population in South Korea, accounting for 20% of all registered individuals with disabilities. The Korea National Disability Registration System (KNDRS), established in 1989, provides social welfare benefits based on medical assessments and predefined disability criteria. As of 2021, 2.645 million individuals (approximately 5.1% of the total population) were registered under this system, covering 15 legally defined disability types, with extremity disabilities being the most common (45.1%) [28].

A two-step modified snowball sampling approach was used to recruit community health and social workers from disability-related organizations. First, with assistance from the Gyeonggi local government and guidance from a multidisciplinary advisory group—comprising three rehabilitation physicians, two nurses, and two social workers with extensive experience working with people with disabilities in Gyeonggi Province (each with 10 to 20 years of experience)—we compiled a list of relevant community health and social care resources. These resources encompassed medical care, mobility support, and caregiving services. Details on each resource and its main services, as described on official websites or in service manuals, are provided in Table 1.

**Table 1:** Community health and social care resources for PWDs and their main services.


COMMUNITY HEALTH AND SOCIAL CARE RESOURCES		MAIN SERVICES

**Medical**	Primary health care teams for PWDs	Primary health care services (medication, treatment, health assessment) Home-based primary care health assessment and planning

	Rehabilitation medical centers (clinic, hospital)	Rehabilitation treatment and medication

	Medical centers (primary, secondary, tertiary hospital)	Primary and special care services (medication, treatment, health assessment)

	Disability-friendly health checkup facilities	Health assessment with disability assistive device (x-ray etc.)

	Dental care centers for the persons with special needs	Dental treatment and medication (including dental anesthesia)

**Public health**	Regional health & medical centers for PWDs	Case management Liaison service, referral to medical and social resources Health education and administration

	Community mental health welfare centers	Mental health care management Mental health education

	Public health centers: community-based rehabilitation teams	Health promotion program Rehabilitation education

	Public health centers: visiting nursing teams	Health promotion program Home-based care (excluding medical care)

**Social welfare**	Social welfare centers for PWDs	Case management Leisure services Advocacy

	Residential homes for PWDs	Housing care services

	Day care centers for PWDs	Day care services

	Vocational rehabilitation facilities for PWDs	Vocational rehabilitation Job referral

	Social service centers (activity assistant or long-term care)	Caregiving services (daily activities)

	Assistive technology service centers	Sales, rental, maintenance, repairs, and training on how to use the assistive technology devices

	Mobility support centers for the transportation vulnerable	Transport services

	Community service centers (local government office)	Administrative services (social service application, disability registration etc.) Case management

	Advocacy agencies for PWDs	Advocating on behalf of victims with disabilities (rights, abuse etc.)

	Family support centers for PWDs	Self-help activities, social networking, leisure programs for families with PWDs

**Disability association**	Associations of PWDs	Social networking, advocacy, social participation programs


Abbreviations. PWDs = People with disabilities.

Next, we directly contacted workers from 20 key community health and social care agencies engaged in intersectoral collaboration with the Gyeonggi RHMC for PWDs. These workers served as seeds for the snowball survey and were invited to participate in a web-based survey via email. In the second step, we asked these staff members to distribute the survey link to workers from other agencies that had partnerships with them and were involved in integrated care for PWDs, further expanding the recruitment pool. A total of 203 participants responded to the survey between November and December 2021.

### Measurement and variables

As an exploratory study examining intersectoral collaboration among disability-related resources in Gyeonggi Province, a stepwise questionnaire was developed. For each community organization related to integrated care for PWDs, respondents were asked to indicate their awareness of the organization as a potential partner, their perceived importance and frequency of collaboration with the organization, and—if they had prior collaboration experience—their satisfaction with that collaboration.

The awareness of each of the 20 listed resources was considered a “yes” response to the question, “Are you aware of this resource as a partner for integrated care for PWDs?” For example, if respondents answered “yes” to the question, “Are you aware of the disability-friendly health checkup facilities?” they were considered aware of these facilities. In the second step, respondents were asked to rate the perceived importance of collaboration with the resource using the question: “From the perspective of integrated care for PWDs in your work, how important do you think collaboration with the disability-friendly health checkup facilities is?” Responses were recorded on a five-point Likert scale, ranging from 1 (not at all important) to 5 (extremely important). Next, the frequency of collaboration was assessed using the question: “How often do you collaborate with the disability-friendly health checkup facilities? (Including information exchange, service provision, referrals, etc.)” Responses were recorded on a five-point Likert scale, ranging from 1 (not at all) to 5 (extremely frequent). Only respondents who selected a response other than “not at all” were asked to rate their satisfaction with the collaboration. Satisfaction was recorded on a five-point Likert scale, ranging from 1 (extremely unsatisfied) to 5 (extremely satisfied).

Additionally, to capture broader perceptions of intersectoral collaboration, an open-ended question was included: “Please provide any suggestions or opinions about intersectoral collaboration with community health and social resources for PWDs.”

### Statistical analysis

We conducted a descriptive analysis to identify the respondents’ organizational affiliation, occupation, and work experience. Data on awareness are presented as numbers and percentages, while importance and frequency scores are expressed as means and standard deviations. The difference between importance and frequency scores was calculated using a paired t-test, and differences based on organizational affiliation were assessed using Welch’s *F* test.

The importance-performance analysis (IPA) method was used to visualize the differences between the importance and frequency of intersectoral collaborations with each community resource. IPA aims to identify attributes for which a product or service underperforms or overperforms and displays them on an easily interpreted two-dimensional grid. The IPA technique for developing marketing strategies has been applied in various fields, including health service provision [29,30]. The Y axis of the IPA grid indicates respondents’ perceived importance of selected attributes, while the x-axis indicates the performance for these attributes. We used mean importance and frequency ratings for the collaboration with each community resource. The four quadrants of the IPA grid and their interpretation are as follows: “Concentrate Here – high importance and low performance,” “Keep up the Good Work – high importance and high performance,” “Low priority – low importance and low performance,” “Possible Overkill – low importance and high performance.”

Additionally, responses to an open-ended question designed to understand community health and social workers’ perceptions were analyzed using a qualitative content analysis method.

### Ethics statement

The study protocol was reviewed and approved by the Institutional Review Board of B Hospital (approval no. B-2111-721-30). The purpose of the survey and informed consent were provided within the web-based survey, and responses were collected via the online survey tool. As the survey was sent to individual workers’ emails, no additional permissions were required from the organizations to which the respondents belonged.

## Results

A total of 203 community health and social care workers from 13 agencies serving PWDs among the 20 listed organizations responded to the survey. Regardless of profession—including social workers, physiotherapists, occupational therapists, and nurses—30.5% of the respondents were affiliated with organizations related to social welfare and community services. The proportions of respondents affiliated with medical facilities and public health agencies were similar (28.6% vs. 27.1%, respectively). In terms of work experience, 39% of respondents had less than three years of experience, while 28.6% had more than ten years of work experience. Further details on the respondent characteristics are presented in Table 2.

**Table 2:** Characteristics of the study populations.


VARIABLES	N (%)

**Total**	**203 (100.0)**

**Work experience in disability-related organizations**	

Less than 1 year	34 (16.75)

1–3 years	45 (22.17)

3–5 years	31 (15.27)

5–10 years	35 (17.24)

More than 10 years	58 (28.57)

**Affiliated organizations**	

Type 1. Medical service (clinics, hospitals, rehabilitation medical centers, dental hospitals)	**58 (28.57)**

Rehabilitation medical centers (clinic, hospital)	16 (7.88)

Medical centers (primary, secondary, tertiary hospital)	42 (20.69)

Type 2. Public health service (public health centers, community-based mental health centers, etc.)	**55 (27.09)**

Community mental health welfare centers	6 (2.96)

Public health centers: community-based rehabilitation teams	13 (6.40)

Public health centers: visiting nursing teams	36 (17.73)

Type 3. Social welfare and community service (social welfare centers, vocational rehabilitation facilities, assistive technology service centers, etc.)	**62 (30.54)**

Social welfare centers for PWDs	38 (18.72)

Residential homes for PWDs	2 (0.99)

Day care centers for PWDs	6 (2.96)

Vocational rehabilitation facilities for PWDs	1 (0.49)

Social service centers (activity assistant or long-term care)	6 (2.96)

Advocacy agencies for PWDs	3 (1.48)

Family support centers for PWDs	6 (2.96)

Type 4. Disability association	**28 (13.79)**

**Background of professionals**	

Physicians	2 (0.99)

Nurses	38 (18.72)

Physiotherapists/Occupational therapists/Exercise Physiologist	21 (10.34)

Social workers	113 (55.67)

Formal caregivers	1 (0.49)

Special educators	1 (0.49)

Administrators/Public servants	19 (9.36)

Technicians (environmental accessibility experts, assistive technology specialists)	3 (1.48)

Disability advocates	5 (2.46)


The participants’ awareness of each community resource varied (Table 3). They were generally more aware of social welfare organizations as key partners for intersectoral collaboration, including social welfare facilities for PWDs, mobility support centers, and local government community service centers. In contrast, their awareness of health-related organizations was relatively low, with the exception of medical facilities such as clinics and hospitals. Notably, only 20–50% of them were aware of the newly introduced disability-focused organizations designated by the Korean government under the Act on Right to Health for PWDs, such as primary healthcare pilot program teams for PWDs, disability-friendly health checkup facilities, and the RHMC for PWDs.

**Table 3:** Awareness of Community Health and Social Care Organizations for PWDs, and Perceived Importance, Frequency, and Satisfaction with Collaboration with These Organizations.


COMMUNITY HEALTH AND SOCIAL RESOURCES (n ^a^)		AWARENESS N (%)	IMPORTANCE (M ± SD)	FREQUENCY (M ± SD)	SATISFACTION (M ± SD)

**Medical**	^b^ Primary health care teams for PWDs (n = 203, 14)	84 (41.38)	4.10 ± 0.82	1.12 ± 0.50	3.50 ± 0.76

	Rehabilitation medical centers (clinic, hospital) (n = 187, 68)	139 (74.33)	4.19 ± 0.79	1.61 ± 0.95	3.38 ± 0.65

	Medical centers (primary, secondary, tertiary hospital) (n = 168, 80)	132 (81.99)	4.14 ± 0.76	1.81 ± 0.98	3.43 ± 0.76

	^b^ Disability-friendly health checkup facilities (n = 203, 7)	43 (21.18)	4.06 ± 0.88	1.04 ± 0.25	3.57 ± 1.13

	Dental care centers for the persons with special needs (n = 203, 26)	76 (37.44)	4.11 ± 0.84	1.22 ± 0.64	3.69 ± 0.74

**Public health**	^b^ Regional health & medical centers for PWDs (n = 203, 57)	107 (52.71)	4.22 ± 0.75	1.53 ± 0.99	3.91 ± 0.83

	Community mental health welfare centers (n =197, 91)	14 (71.07)	4.18 ± 0.86	1.75 ± 0.97	3.45 ± 0.75

	Public health centers: community-based rehabilitation teams (n = 190, 41)	86 (45.26)	4.22 ± 0.84	1.40 ± 0.85	3.61 ± 0.70

	Public health centers: visiting nursing teams (n = 167, 65)	116 (69.46)	4.24 ± 0.81	1.63 ± 0.97	3.52 ± 0.92

**Social welfare**	Social welfare centers for PWDs (n = 165, 143)	148 (89.70)	4.32 ± 0.75	2.75 ± 1.14	3.65 ± 0.77

	Residential homes for PWDs (n = 201, 79)	161 (80.10)	4.10 ± 0.83	1.67 ± 0.97	3.54 ± 0.66

	Day care centers for PWDs (n = 197, 83)	152 (77.16)	4.14 ± 0.80	1.77 ± 1.06	3.59 ± 0.66

	Vocational rehabilitation facilities for PWDs (n = 202, 81)	157 (77.72)	4.17 ± 0.78	1.72 ± 1.04	3.67 ± 0.67

	Social service centers (activity assistant or long-term care) (n = 197, 115)	159 (80.71)	4.33 ± 0.74	2.38 ± 1.43	3.65 ± 0.66

	Assistive technology service centers (n = 203, 85)	145 (71.43)	4.25 ± 0.78	1.93 ± 1.29	3.67 ± 0.71

	Mobility support centers for the transportation vulnerable (n = 203, 99)	165 (81.28)	4.32 ± 0.83	2.09 ± 1.33	3.63 ± 0.78

	Community service centers (n = 203, 141)	176 (86.70)	4.40 ± 0.75	2.71 ± 1.43	3.52 ± 0.74

	Advocacy agencies for PWDs (n = 200, 49)	106 (53.00)	4.00 ± 0.84	1.45 ± 0.89	3.76 ± 0.72

	Family support centers for PWDs (n = 197, 47)	129 (65.48)	4.15 ± 0.84	1.41 ± 0.81	3.49 ± 0.62

**Disability association**	Associations of PWDs (n = 175, 65)	135 (77.14)	3.89 ± 0.92	1.73 ± 1.11	3.48 ± 0.75


^a^ The total number of respondents for awareness, importance, and frequency of collaboration with each community resource. Respondents skipped these questions for their own affiliated organization. Satisfaction was only assessed among those with actual collaboration experience. For example, excluding 16 respondents affiliated with rehabilitation medical centers, 187 answered questions on awareness, importance, and frequency of collaboration with these centers, and 68 rated their satisfaction. ^b^ The organizations newly established as part of a policy initiative designated by the Korean government under the Act on Right to Health for PWDs. Abbreviations. PWDs = People with disabilities, N = number of respondents, M = Mean, SD = standard deviation.

Nevertheless, respondents generally perceived intersectoral collaboration with all community health and social care resources as important. Perceived importance scores for collaboration with each resource were consistently above 4 on a 5-point scale, except for collaboration with associations of PWDs. However, despite the high perceived importance, respondents reported relatively infrequent collaboration with these resources, with frequency scores falling below 3 on the same scale (Table 3). Despite the overall low frequency of collaboration, respondents who had actual collaboration experience reported relatively high satisfaction levels, with satisfaction scores ranging from 3.38 to 3.91 on a 5-point scale. A statistically significant discrepancy between the perceived importance and frequency of collaboration was identified for all resources (P < 0.001).

**Figure 1:**
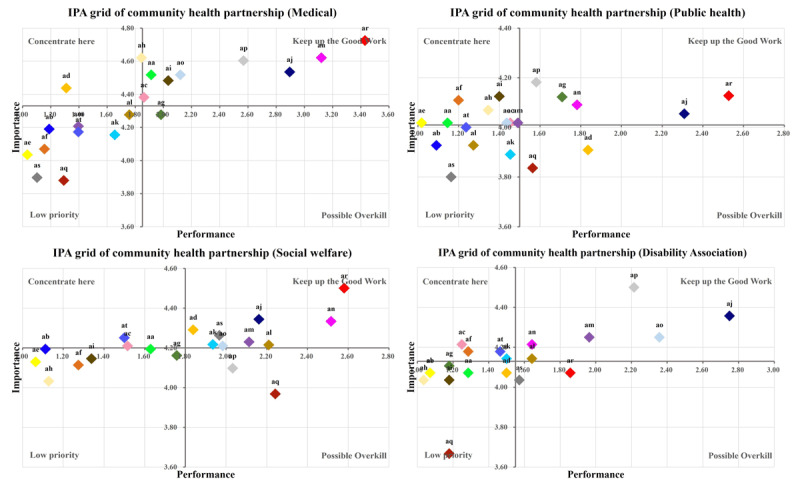
IPA grid of community health partnership according to respondents’ organizational affiliation. aa: Regional health & medical centers for PWDs; ab: Primary health care teams for PWDs; ac: Rehabilitation medical centers; ad: Medical centers; ae: Disability-friendly health checkup facilities; af: Dental care centers for the persons with special needs; ag: Community mental health welfare centers; ah: Public health centers-CBR team; ai: Public health centers-visiting nursing; aj: Social welfare centers for PWDs; ak: Residential homes for PWDs; al: Day care centers for PWDs, am: Vocational rehabilitation facilities for PWDs; an: Social service centers (activity-supporting, long-term care); ao: Assistive technology service centers; ap: Mobility support centers for the transportation vulnerable; aq: Associations of PWDs; ar: Community service centers, as: Advocacy agencies for PWDs; at: Family support centers for PWDs.

Each community resource was plotted based on the mean scores of participants’ perceived importance and frequency of collaboration. The importance of collaboration with each resource is shown on the vertical axis (ranging from low at the bottom to high at the top), while the frequency of collaboration is shown on the horizontal axis (ranging from low on the left to high on the right) (Figure 1). Differences in perceived importance and actual frequency of collaboration emerged based on the types of organizations to which participants were affiliated. Respondents from medical centers or public health organizations generally reported less frequent collaboration with community resources compared to those from social welfare facilities.

In this study, we focused on the “Concentrate Here” and “Low Priority” quadrants of the IPA grid, rather than the “Keep up the Good Work” and “Possible Overkill” quadrants. The “Concentrate Here” quadrant indicates resources that were perceived as important for collaboration but were not frequently engaged with. The “Low Priority” quadrant, while reflecting lower perceived importance, was also analyzed to identify differences in perceived importance based on organizational affiliation.

Table 4 categorizes the community resources into these two quadrants. In both, we identified resources with particularly low levels of collaboration. Participants from medical or public health organizations viewed collaboration with medical and public health-related resources, which were placed in the “Concentrate Here” quadrant, as essential. In contrast, participants from social welfare facilities or associations for PWDs tended to view these resources as less critical, placing them in the “Low Priority” quadrant.

**Table 4:** Community resources on the “Concentrate here (high importance-low performance)” and “Low priority (low importance-low performance)” quadrants of IPA grid by respondents’ organizational affiliation types.


AFFILIATION TYPE	CONCENTRATE HERE	LOW PRIORITY

Medical	Medical centers (m) ^c^ Public health centers-CBR team (p) ^d^	Day care centers for PWDs (s) ^e^ Vocational rehabilitation facilities for PWDs (s) ^b^ Primary health care teams for PWDs (m) Family support centers for PWDs (s) Residential homes for PWDs (s) Dental care centers for the persons with special needs (m) ^b^ Disability-friendly health checkup facilities (m) Advocacy agencies for PWDs (s) Associations of PWDs (d) ^f^

Public-health	Public health centers-visiting nursing (p) Dental care centers for the persons with special needs (m) Public health centers-CBR team (p) ^b^ Regional health & medical center for PWDs (p) Rehabilitation medical centers (m) Assistive technology service centers (s) Vocational rehabilitation facilities for PWDs (s) ^b^ Disability-friendly health checkup facilities (m)	Family support centers for PWDs (s) ^b^ Primary health care teams for PWDs (m) Day care centers for PWDs (s) Residential homes for PWDs (s) Advocacy agencies for PWDs (s)

Social-welfare	Family support centers for PWDs (s) Rehabilitation medical centers (m)	^b^ Primary health care teams for PWDs (m) ^b^ Regional health & medical center for PWDs (p) Community mental health welfare centers (p) ^b^ Disability-friendly health checkup facilities (m) Public health centers-visiting nursing (p) Dental care centers for the persons with special needs (m) Public health centers-CBR team (p)

Disability-association	Rehabilitation medical centers (m) Dental care centers for the persons with special needs (m) Family support centers for PWDs (s) Residential homes for PWDs (s)	Community mental health welfare centers (p) ^b^ Primary health care teams for PWDs (m) ^b^ Disability-friendly health checkup facilities (m) ^b^ Regional health & medical center for PWDs (p) Medical centers (m) Public health centers-CBR team (p) Public health centers-visiting nursing (p) Associations of PWDs (d)


^b^ The organizations newly established as part of a policy initiative by Korean government under the Act on Right to Health for PWDs. ^c^ (m). The community resources were categorized as medical facilities. ^d^ (p). The community resources were categorized as public health agencies. ^e^ (s). The community resources were categorized as social welfare and social service organizations. ^f^ (d). The community resources were categorized as disability associations.

Among participants from medical facilities, the discrepancies between perceived importance and actual frequency of collaboration were significantly greater for associations for PWDs (P = 0.021), vocational rehabilitation facilities (P = 0.010), and daycare centers for PWDs (P = 0.005) compared to those from social welfare facilities. On the other hand, resources such as social service centers (assisting daily activities and long-term care), social welfare centers, mobility support centers for transportation-vulnerable individuals, and community service centers operated by local governments were placed in the “Keep up the Good Work” quadrant. This indicates that, regardless of organizational affiliation, participants consistently viewed these resources as important and reported frequent collaboration with them.

Across all organizational types, disability-focused organizations newly designated under the Act on Right to Health for PWDs were consistently positioned in either the “Concentrate Here” or “Low Priority” quadrants. This suggests infrequent collaboration with these organizations, regardless of whether they were perceived as important or not.

Among respondents, 70 community health and social workers from disability-related organizations (response rate: 34%) provided answers to the open-ended question on intersectoral collaboration. The responses were categorized into six key themes: ‘Information’, ‘Governance or systems’, ‘Administrative process’, ‘Workforce’, ‘Resources’, and ‘Clients’ (Table 5).

**Table 5:** Perceptions on intersectoral collaboration from an open-ended question.


CATEGORIES	N (%)

Total respondents	70 (100.0)

**Information**	37 (52.86)

1. Lack of information about community resources available for collaboration *“Intersectoral collaboration is sometimes hindered due to a lack of awareness about how to engage with other agencies or what services are available for collaboration.” (Id 59)*	21

2. Absence of standardized guidelines and materials explaining services provided by each organization, including practical consulting or counseling guidance for inter-agency collaboration *“I think there is a need for materials that can provide detailed information on the services a client can receive at each collaborating organization during inter-agency collaboration.” (Id 294)*	13

3. Information sharing platforms *“I believe that if a platform is established where information about the services provided by each organization can be shared, it will help prevent the duplication of services and enable more efficient service delivery.” (Id 281)*	3

**Governance or Systems**	18 (25.71)

1. Control tower, central hub, unified delivery system *“Inter-agency collaboration is often carried out in a fragmented manner, resulting in gaps when establishing local networks. A hub organization that takes a leading role in coordinating and maintaining these networks would be beneficial.” (Id 75)*	7

2. Building a formal network system, referral system *“There is a need to establish a formal system that enables seamless collaboration between the welfare and healthcare sectors. This would ensure that when a person with a disability is identified through welfare services, they can also be appropriately referred to and managed by relevant healthcare services.” (Id 159)* *“Establishing formal networks among community organizations can enhance communication among professionals. Over-reliance on informal networks, such as personal connections, risks weakening collaboration when the responsible personnel change.” (Id 64)*	11

**Administrative process**	12 (17.14)

1. Complex and various procedures across the organizations *“It seems that the procedures for inter-agency collaboration are complex, involve a large amount of paperwork, and differ between organizations.” (Id 216)*	11

2. Long waiting times *“When referring clients to public health center services, the long waiting times often make collaboration difficult.” (Id 312)*	1

**Workforce**	22 (31.43)

1. Staff shortage & turnover in collaboration roles *“There is a shortage of staff specifically assigned to manage inter-agency collaboration. (…) Frequent staff turnover prevents continuous connections and makes information transfer challenging.” (Id 118)*	5

2. Staff competency and performance variability *“There are differences in performance in collaboration between organizations depending on the job competencies of workers in intersectoral collaboration.” (Id 10)* *“It is necessary to regularly provide training to disability-related organizations on new government programs aimed at promoting the health rights and health improvement of people with disabilities, including introductions to these programs and guidance on how to access them.” (Id 30)*	4

3. Uncooperative or passive attitudes of staff *“The uncooperative, passive attitude of some partner organizations makes intersectoral collaboration difficult. There was an experience where a referral was made to a mental health welfare center for a person with a developmental disability, but the referral was declined, as they stated that counseling was difficult.” (Id 38)*	13

**Resources**	15 (21.43)

1. Shortage of available service resources *“There seems to be an overall shortage of social services and local resources for rehabilitation services available for PWDs.” (Id 317)*	13

2. Limitations in financial and administrative support for intersectoral collaboration *“When there are no additional incentives for collaboration, many organizations tend to respond passively.” (Id 231)*	2

**Clients**	5 (07.14)

1. Sensitive to sharing information *“When referring a client for inter-agency collaboration, the client is often sensitive about sharing their personal information.” (Id 2)*	2

2. Difficulties in explanation and persuasion *“There is a tendency to rely heavily on the opinions of the client’s caregivers or representatives when determining the necessary services and appropriate organizations. As a result, professional opinions are often overlooked. It is challenging to obtain the client’s consent for inter-agency collaboration.” (Id 5)*	3


Notably, the theme of ‘Information’ emerged most prominently from the data. Respondents highlighted that the lack of information about available disability-related community resources, along with the absence of sufficient guidelines and training on inter-agency collaboration, posed significant barriers to effective collaboration. Some respondents also suggested that establishing information-sharing platforms could enhance collaboration across agencies.

Regarding ‘Governance or Systems,’ respondents pointed out that fragmented health and social systems hinder effective collaboration. To address this, some proposed the establishment of a unified delivery system or the designation of a hub organization responsible for coordinating and maintaining intersectoral collaboration networks. Additionally, rather than relying on informal networks, respondents recommended building formal network systems and establishing effective referral systems among relevant organizations.

In terms of the ‘Administrative Process,’ respondents identified complex and varied procedures across organizations as burdensome to collaboration. Regarding the ‘Workforce,’ respondents pointed out challenges such as the shortage and frequent turnover of staff responsible for inter-agency collaboration, inadequate staff competencies in collaboration or understanding disability-related characteristics, and uncooperative attitudes as barriers to successful collaboration.

Respondents also noted difficulties stemming from the lack of available community resources, particularly in areas like rehabilitation services, as well as limitations in financial and administrative support for intersectoral collaboration. Finally, under the ‘Client’ theme, respondents highlighted the challenges of obtaining client consent to share their information with other organizations, as well as the difficulty in explaining or persuading clients of the necessity of intersectoral collaboration.

## Discussion

This explanatory study aimed to examine: 1) the awareness, collaboration, and satisfaction levels of community health and social workers from disability-related organizations, and 2) their perceptions of intersectoral collaboration. We assessed the extent to which these workers recognize community resources as integrated care partners. Using the IPA method, we highlighted the discrepancy between the perceived importance of and actual frequency of intersectoral collaboration with these community resources. Our findings revealed that, compared to social sector organizations, there was relatively low awareness of medical and public health sector agencies, particularly the newly designated disability-focused organizations introduced by the Korean government. Community health and social workers reported infrequent cross-sectoral cooperation despite recognizing its importance. Although collaboration was infrequent, workers who had experience collaborating with each community organization expressed high levels of satisfaction with these partnerships. The key findings of this study—including the differences and commonalities in the perceived importance and frequency of collaboration across sectors, as well as deeper insights from the qualitative results—present valuable opportunities for policymakers to implement strategies that foster cross-sectoral partnerships, ultimately enhancing the health and well-being of PWDs.

Awareness of services and policies is a prerequisite for action, whether for service utilization or inter-agency cooperation [31], and this is particularly important in the context of newly introduced initiatives. This aligns with our qualitative finding that respondents emphasized the necessity of detailed *information* about available community resources, especially regarding those in different professional sectors. Notably, the poor awareness of disability-friendly agencies—such as the primary healthcare pilot program teams and the RHMC under the *Act on Right to Health for PWDs*. in Korea—can be interpreted in two ways. First, although five years have passed since the introduction of these disability-inclusive initiatives, the workers’ low awareness may have contributed to the infrequent collaboration with these agencies. Second, this poor awareness may also have influenced respondents’ low perceived importance of collaborating with these agencies, stemming from a lack of mutual understanding of each sector’s roles and responsibilities, ultimately placing these agencies in the ‘Low Priority’ quadrant of the IPA grid. Collaboration is likely to deteriorate when there is no shared understanding of each other’s roles, and workers may resist efforts to establish partnerships if their roles and responsibilities are unclear [18]. A previous study on the newly implemented disability-focused primary healthcare project also highlighted this low awareness, noting that no opportunity was provided to assess the project’s effectiveness from the perspective of consumers before the government’s formal evaluation took place [25]. This suggests that policymakers and service providers involved in these new government-funded, disability-inclusive services could enhance awareness by actively promoting their roles and operational processes through targeted publicity campaigns and continuous training programs focusing on available disability-related community resources. However, this study had limitations in capturing deeper insights into the factors influencing perceived importance and necessity for collaboration with each resource. Therefore, future research exploring community health and social workers’ perceptions of these newly introduced disability-friendly agencies could provide a more comprehensive understanding.

Action for cooperation is shaped not only by workers’ awareness and perceived importance, but also by systemic and external barriers, such as incompatible organizational structures and resource constraints [14,17]. Our findings—showing low frequency of collaboration with disability-friendly agencies despite high perceived importance—combined with qualitative results, such as ‘limited financial and administrative support for intersectoral collaboration’ and ‘staff shortages and low competency,’ suggest the presence of systemic barriers to effective cross-sectoral collaboration. The inadequate supply structure of primary healthcare pilot program teams for PWDs has already been identified as a barrier to the full implementation of this pilot project in previous research [25]. Furthermore, insufficient organizational resources can hinder intersectoral collaboration. Managers, such as care coordinators, who invest time in developing partnerships and maintaining communication with community partners, play a critical role in facilitating collaboration across sectors [17]. However, most newly introduced disability-friendly agencies—particularly primary healthcare pilot program teams and disability-friendly medical checkup facilities—are unable to hire dedicated staff to focus on community partnerships due to financial constraints. Instead, these agencies primarily employ physicians and nurses, whose main focus is on delivering medical care. Consequently, care coordination and community health partnerships are often deprioritized due to their heavy workloads and insufficient staffing.

Community health and social workers from a medical or public health organization recognized that building partnerships with medical or public health community resources was essential and that social welfare types of community resources were less critical. Conversely, service providers working for social welfare or associations of PWDs recognized the resources of either healthcare or public health types as less important. It could be supposed that community health and social workers for PWDs require partnerships with similar sectors to accomplish their goals based on a shared understanding of roles and values, as well as complex administrative processes and insufficient incentives in intersectoral collaboration. It is consistent with a prior study on perceptions of healthcare alliances, which found that collaboration with nonmedical health sectors, such as public health and behavioral health, was more important than collaboration with non-health sectors, such as transportation and housing [16]. Nonetheless, disability policies and practices have emphasized an intersectoral collaborative and coordinated approach, with the evidence that cross-sectoral collaboration, including housing and education, can reduce the use of hospitals and nursing homes for PWDs and improve their general health and participation in the community [19]. Thus, disability training for the disability-competent workforce should highlight the importance of identifying care coordination needs across the spectrum of services, managing care transitions, and leveraging community support. This can help community health and social workers shift from viewing disability as an illness to be prevented or treated to seeing disability in terms of functional limitations that may or may not limit an individual’s health and quality of life [32].

Regardless of organizational affiliation, common and shared values were also identified in our study. Community health and social workers perceived the high importance of cooperation with social service centers (daily activity supporting, long-term care), social welfare centers, mobility support centers for the transportation vulnerable, and local governments’ community service centers, which were positioned on the “Keep up the Good Work” quadrant, and frequently collaborated as well. One interpretation of these findings is that there is a consensus on the necessity of caregiving and transport support issues in South Korea. Community health and social workers perceived local governments’ community service centers as the most valuable resource and collaborated with them. This can be interpreted as two enablers of intersectoral collaboration, already mentioned in various studies: sharing information and a single entry point [17,18,22]. Under the Korea National Disability Registration System (KNDRS), community service centers run by the local government have authority over disability-related information. The KNDRS, having focused on a medical or impairment approach, has limitations for delivering personalized services without reflecting other aspects of disability, such as socioeconomic and functional status. Nonetheless, it is used to deliver most public or social services to PWDs with the benefits of objective measures to assess disability and convenient means that can be linked to national health data [33]. Additionally, the centers have been a single entry point for applying for most public or social services and disability registration.

This study has several limitations. First, selection bias may exist because data were collected using a modified snowball sampling approach within the context of the Korean disability sector, despite our efforts to recruit diverse community agencies. As a result, the interpretation and generalizability of the findings should be approached with caution. Second, although educational and housing-related agencies play critical roles in the health and well-being of PWDs, they were omitted from our measurement of community health and social care resources. Third, this study focused on examining community health and social workers’ awareness, perceived importance, frequency, and satisfaction with collaboration involving community resources. Therefore, we were unable to capture deeper qualitative insights into the specific facilitators or barriers to intersectoral collaboration with each type of resource. Lastly, although the perspectives of people with lived experience are essential for developing disability-inclusive policies, this study focused only on the perspectives of service providers.

As cross-sectoral collaboration and disability-inclusive policymaking gain increasing importance, further research should explore the enablers and barriers to effective intersectoral collaboration, with an emphasis on understanding the roles, responsibilities, and shared goals across sectors, including the perspectives of PWDs.

## Conclusions

Although collaborative action is inherently challenging due to the broad spectrum of health and health-related determinants associated with disability, cross-sectoral collaboration—including medical, public health, social welfare, and disability advocacy organizations—is essential for promoting the health and well-being of PWDs. This study is the first to examine community health and social workers’ perceptions of intersectoral collaboration with community resources, including newly established disability-focused agencies, under South Korea’s disability-inclusive initiatives. We highlighted perceptual factors that may promote or hinder cross-sectoral collaboration, particularly workers’ awareness of community resources as collaborative partners and their perceived importance of collaborating with these resources. Our findings suggest that awareness and perceived importance can be seen as seeds for fostering collaboration, highlighting the value of future research to explore how these initial perceptions may grow into actual collaborative practices. Furthermore, this study identified both differences and commonalities in perceived importance and frequency of collaboration across different sectors, which were systematically visualized using the IPA grid. These differences may reflect each sector’s unique service mandates and target beneficiaries. However, the particularly low collaboration frequency with some sectors—despite their high perceived importance—warrants further investigation, particularly into persistent structural and cultural barriers between the health and social sectors, which have been long-standing issues in the disability field.

From a governance and systems perspective, establishing a unified service delivery mechanism or formally designating a coordinating hub organization could foster sustainable intersectoral collaboration. In South Korea, RHMCs—which were introduced to bridge the gap between community healthcare and social care for PWDs—could be well-positioned to play this coordinating role. As a starting point, RHMCs could take responsibility for identifying available community resources, training relevant professionals on collaborative processes, and organizing formal networks to cultivate mutual understanding and shared goals for improving the health and well-being of PWDs. Additionally, information-sharing systems and financial incentives—such as targeted funding or collaboration bonuses—could further enhance the feasibility and sustainability of intersectoral collaboration.
